# Health, nutrition, and development of children born preterm and low birth weight in rural Rwanda: a cross-sectional study

**DOI:** 10.1186/s12887-017-0946-1

**Published:** 2017-11-15

**Authors:** Catherine M. Kirk, Jean Claude Uwamungu, Kim Wilson, Bethany L. Hedt-Gauthier, Neo Tapela, Peter Niyigena, Christian Rusangwa, Merab Nyishime, Evrard Nahimana, Fulgence Nkikabahizi, Christine Mutaganzwa, Eric Ngabireyimana, Francis Mutabazi, Hema Magge

**Affiliations:** 1 Partners In Health/Inshuti Mu Buzima, Rwinkwavu, Rwanda; 2 Division of General Pediatrics, Boston Children’s Hospital, Boston, MA USA; 3 Department of Global Health and Social Medicine, Harvard Medical School, Boston, MA USA; 4 Division of Global Health Equity, Brigham and Women’s Hospital, Boston, MA USA; 5 Rwinkwavu District Hospital, Ministry of Health, Rwinkwavu, Rwanda

**Keywords:** Prematurity, Low birth weight, Nutrition, Early childhood development, Sub-Saharan Africa

## Abstract

**Background:**

As care for preterm and low birth weight (LBW) infants improves in resource-limited settings, more infants are surviving the neonatal period. Preterm and (LBW) infants are at high-risk of nutritional and medical comorbidities, yet little is known about their developmental outcomes in low-income countries. This study evaluated the health, nutritional, and developmental status of preterm/LBW children at ages 1–3 years in Rwanda.

**Methods:**

Cross-sectional study of preterm/LBW infants discharged between October 2011 and October 2013 from a hospital neonatal unit in rural Rwanda. Gestational age and birth weight were gathered from hospital records to classify small for gestational age (SGA) at birth and prematurity. Children were located in the community for household assessments in November–December 2014. Caregivers reported demographics, health status, and child development using locally-adapted Ages and Stages Questionnaires (ASQ-3). Anthropometrics were measured. Bivariate associations with continuous ASQ-3 scores were conducted using Wilcoxon Rank Sum and Kruskal Wallis tests.

**Results:**

Of 158 eligible preterm/LBW children discharged from the neonatal unit, 86 (54.4%) were alive and located for follow-up. Median birth weight was 1650 grams, median gestational age was 33 weeks, and 50.5% were SGA at birth. At the time of household interviews, median age was 22.5 months, 46.5% of children had feeding difficulties and 39.5% reported signs of anemia. 78.3% of children were stunted and 8.8% wasted. 67.4% had abnormal developmental screening. Feeding difficulties (*p* = 0.008), anemia symptoms (*p* = 0.040), microcephaly (*p* = 0.004), stunting (*p* = 0.034), SGA (*p* = 0.023), very LBW (*p* = 0.043), lower caregiver education (*p* = 0.001), and more children in the household (*p* = 0.016) were associated with lower ASQ-3 scores.

**Conclusions:**

High levels of health, growth, and developmental abnormalities were seen in preterm/LBW children at age 1–3 years. As we achieve necessary gains in newborn survival in resource-limited settings, follow-up and early intervention services are critical for ensuring high-risk children reach their developmental potential.

**Electronic supplementary material:**

The online version of this article doi: (10.1186/s12887-017-0946-1) contains supplementary material, which is available to authorized users.

## Background

Children in resource-limited settings are failing to reach their developmental potential due to widespread adversities such as poverty and malnutrition [1]. Children facing additional perinatal risk factors, such as prematurity, low birth weight (LBW), and intrauterine growth restriction (IUGR), are at greater risk of dying in childhood [2, 3]. Preterm and LBW (PT/LBW) infants who survive are likely to face additional developmental challenges [4–6] and comorbidities such as respiratory disease [7], feeding difficulties [8], and malnutrition [9]. In high-income countries, longitudinal follow-up and early intervention for at-risk infants are standard care [10]; however, such standard services do not exist in resource-limited settings.

Globally, prematurity-related complications are the leading cause of death among neonates and children [11]. For preterm infants who survive the neonatal period, data from resource-limited countries on their long-term outcomes are limited [12]. The little evidence from middle-income countries shows worse growth [13] and developmental outcomes for PT/LBW children [14, 15]. As neonatal survival interventions are implemented, efforts to understand and improve their long-term outcomes and quality of life become even more critical.

Rwanda has recorded dramatic declines in child mortality [16], however, neonatal deaths account for 40% of Rwanda’s under-five deaths [17] and prematurity is one of the leading causes of child mortality [18]. The Government of Rwanda has prioritized newborn survival initiatives and developed a national protocol for the care of sick and small newborns. Partners In Health-Inshuti Mu Buzima (PIH/IMB) has supported Rwanda’s Ministry of Health to strengthen the health system since 2005 including focused interventions to improve newborn survival. This study aims to assess the health, nutrition, and development of children born PT/LBW who were discharged from a district hospital neonatal care unit in rural Rwanda. It was hypothesized that children born PT/LBW would have impaired developmental outcomes.

## Methods

This study includes a cross-section of children born PT/LBW discharged from the neonatal unit at Rwinkwavu District Hospital between October 2011 and October 2013. Rwinkwavu District Hospital in rural Southern Kayonza district serves nearly 200,000 people [19] and is supported by PIH/IMB. The neonatal unit at Rwinkwavu District Hospital opened in 2010 and is staffed by a general practitioner physician and nurses. The unit can provide basic specialized newborn care including kangaroo mother care, incubator management, oxygen therapy, intravenous fluids, phototherapy, and specialized feeding protocols.

### Participants

Children discharged between October 2011 and October 2013 were identified from patient registers and included if they were born preterm (documented gestational age < 37 weeks or recorded as preterm) or with a documented birth weight of <2000 g. Children were excluded if they were term and missing a documented birth weight or if they had documented genetic dysmorphologies, congenital heart disease, birth asphyxia, or died prior to discharge. Patient charts were reviewed to verify gestational age or prematurity status, birth weight, and absence of exclusion criteria.

Household data collection took place from November to December 2014. Community health workers helped identify households based on the geographic location and the caregiver’s name from hospital records. Then, a team of trained data collectors invited the child’s primary caregiver to participate in the study. Consenting caregivers were interviewed in their home and data was collected using Android tablets. Primary caregivers reported on household demographics, the child’s health and development, and direct anthropometric assessments were completed.

### Measures

Clinical data included birth weight, gestational age, age at admission to the neonatal unit, and duration of stay in the neonatal unit. Birth weight and gestational age were categorized using World Health Organization (WHO) guidelines as follows: LBW (<2500 g), very LBW (VLBW; <1500 g), and extremely LBW (ELBW; <1000 g). Gestational age was categorizes as: term (gestational age ≥ 37 weeks), moderate to late preterm (32 to <37 weeks), and very preterm (<32 weeks). Children were assessed for size at birth if they had both birth weight and gestational age documented. Children were defined as small for gestational age (SGA) if below the 10th percentile in weight for gestational age using INTERGROWTH-21st preterm growth standards for size at birth [20, 21].

Children were grouped into three categories based on their age at admission: day of birth (0 days), 1 day after birth, and greater than 1 day after birth. The Rwandan community-based ranking system for poverty known as *ubudehe* measured socioeconomic status; *Ubudehe* uses poverty categories that range from one to six with one being the destitute poor and six being the most well-off [22].

Head circumference was measured using a tape measure and assessed (microcephaly or macrocephaly) using WHO Growth Standards [23]. Caregivers reported on the child’s health status by responding to questions about whether or not the child experienced any symptoms of common conditions among children born PT/LBW (non-copyrighted measures are available in the online Additional file 1). Caregivers were asked if the child showed signs of anemia (pale, weak, or history of transfusions), feeding difficulties (choking, coughing, or gagging), or respiratory disease (difficulty breathing on a daily basis such as fast breathing, cough, or out of breath from walking). This method of caregiver-report of symptoms in young children has been used in other research to study both chronic and acute conditions [24, 25].

Nutrition data were collected using standard anthropometric procedures. Two trained data collectors measured the child’s length/height and weight. Nutritional outcomes were scored using WHO Growth Standards [26]. Stunting (low height/length-for-age), wasting (low weight-for-height/length), and underweight (low weight-for-age) were defined as moderate or severe if z-scores were 2 or 3 standard deviations below the mean, respectively.

The Ages and Stages Questionnaires (ASQ-3), a screening measure used in Rwanda and other sub-Saharan Africa countries [27–30] measured children’s development. Caregivers answered 30 age-specific questions covering five domains of development (communication, gross motor, fine motor, problem solving, and personal-social skills). Eleven age-specific ASQ-3 forms covering children ages 12–36 months were used. The ASQ-3 was previously adapted to the Rwandan context, translated into Kinyarwanda, and piloted for caregiver comprehension [31]. The ASQ-3 was scored as a continuous outcome by summing individual items (maximum score of 300), as well as categorically by developmental status (on-track, in the monitoring zone for potential concern, or below cut-off based on falling below standardized cut points in any one domain on the age-specific form) [32]. Chronologic age was used for assessments rather than age adjusted for prematurity as gestational age was not available for all children.

### Analysis

Descriptive statistics were calculated. Fisher’s exact tests (categorical variables) and Kruskal-Wallis or Wilcoxon Rank Sum tests (continuous variables) were used to assess differences in baseline characteristics among children located and those lost to follow-up and differences in health, nutritional, and developmental status by age. Factors associated with continuous scores on the ASQ-3 were assessed using Wilcoxon Rank Sum and Kruskal Wallis tests. Stata 14 (StataCorp, College Station, TX) was used for analyses.

### Ethics

Written informed consent was obtained from all caregivers for themself and their child. The Rwanda National Ethics Committee, Boston Children’s Hospital’s Institutional Review Board, and Rwandan Ministry of Health approved the study.

## Results

Of 201 children discharged from Rwinkwavu District Hospital’s neonatal unit between October 2011 and October 2013, 158 (78.6%) children met study inclusion criteria. Of the 158 eligible children, 54.4% (*n* = 86) were alive and located for household data collection. No caregivers refused to participate; however, 39.9% (*n* = 63) could not be found because the household was unknown (*n* = 48) or relocated outside of Southern Kayonza district (*n* = 15). Six percent (*n* = 9) were located but had died between discharge and household data collection.

Of the 158 eligible children, 46.2% (*n* = 73) were males (Table 1). Median birth weight was 1650 g (interquartile range (IQR): 1450–1850 g) and 25.8% (*n* = 40) were either VLBW or ELBW. Of the 94 (59.5%) infants with gestational age recorded, 64.9% (*n* = 61) were moderate/late preterm and 24.5% (*n* = 23) were very preterm. Half of children assessed for size at birth were SGA (50.5%, *n* = 47/93). Prematurity was the only factor that was significantly different between children who were assessed in household data collection and those who were not located or dead (*p* = 0.025).

**Table 1:** Descriptive characteristics of children discharged from the neonatal unit

	Overall	Alive and assessed	Lost to follow-up	Died between discharge and follow-up	*p*-value
Total number of children, N (%)	158 (100)	86 (54.4)	63 (39.9)	9 (5.7)	
Male gender, n (%)	73 (46.2)	42 (48.8)	29 (46.0)	2 (22.2)	0.320
Birth weight in grams, median (IQR)	1650 (1450–1850)	1650 (1500–1850)	1700 (1420–1900)	1500 (1300–1600)	0.124
Birth weight Status (*n* = 155), n (%)					0.106
Extremely low birth weight (ELBW)	3 (1.9)	0	3 (4.8)	0	
Very low birth weight (VLBW)	37 (23.9)	17 (20.5)	16 (25.4)	4 (44.4)	
Low birth weight (LBW)	115 (74.2)	66 (79.5)	44 (69.8)	5 (55.6)	
Gestational age (*n* = 94), median (IQR)	33 (32–35)	33 (32–35)	33 (31–34)	31 (30–33)	0.060
Prematurity Category (*n* = 94), n (%)					0.025
Very preterm (<32 weeks)	23 (24.5)	7 (12.7)	13 (39.4)	3 (50.0)	
Moderate/late preterm (32 to <37 weeks)	61 (64.9)	41 (74.6)	17 (51.5)	3 (50.0)	
Term (> = 37 weeks)	10 (10.6)	7 (12.7)	3 (9.1)	0	
Size at Birth (*n* = 93), n (%)					1.128
Small for gestational age	47 (50.5)	32 (59.3)	13 (39.4)	2 (33.3)	
Average for gestational age	46 (49.5)	22 (40.7)	20 (60.6)	4 (66.7)	
Overall risk category (*n* = 93), n (%)					0.391
Term and small for gestational age	9 (9.7)	7 (13.0)	2 (6.1)	0	
Preterm and small for gestational age	38 (40.9)	25 (46.3)	11 (33.3)	2 (33.3)	
Preterm and not small for gestational age	46 (49.6)	22 (40.7)	20 (60.6)	4 (66.7)	
Age at admission, n (%)					0.452
0 days, n (%)	121 (78.1)	66 (78.6)	48 (77.4)	7 (77.8)	
1 day, n (%)	18 (11.6)	12 (14.3)	5 (8.1)	1 (11.1)	
> 1 day, n (%)	16 (10.3)	6 (7.1)	9 (14.5)	1 (11.1)	
Days spent in neonatal unit, median (IQR)	12 (5–21)	12 (5–19)	13 (4–21)	23 (11–29)	0.154

The median age of children at household assessment was 22.5 months (IQR: 17.5–30.5) (Table 2). The median number of children in the household, including the assessed child, was three (IQR: 2–5). Only 81.4% (*n* = 70) of households knew their *ubudehe* status; of these, all were relatively poor with 22.8% (*n* = 16) and 77.1% (*n* = 54) of caregivers in category two (very poor) and three (poor), respectively. Nearly all reporting caregivers were the child’s mother (95.4%, *n* = 82); 22.1% (*n* = 19) of caregivers had no formal education and 69.6% (*n* = 59) completed primary school.

**Table 2:** Sociodemographic characteristics of children alive and assessed

Age (in months), median (IQR)	22.5 (17.5–30.5)
Number of children in the household, median (IQR)	3 (2–5)
Number of other children in the household, n (%)	
No other children in the household	18 (20.9)
1–3 other children in the household	44 (51.2)
4 or more other children in the household	24 (27.9)
Caregiver’s Socioeconomic status (SES), n (%)	
Category 2	16 (18.6)
Category 3	54 (62.8)
Unknown category	16 (18.6)
Caregiver’s relationship to child, n (%)	
Mother	82 (95.4)
Other	4 (4.7)
Caregiver’s highest education level, n (%)	
None	19 (22.1)
Primary	59 (69.6)
Secondary	8 (9.3)

Abnormal head circumference was seen in 9.6% of children, with 6.0% (*n* = 5/84) meeting criteria for microcephaly and 3.6% (*n* = 3/84) for macrocephaly (Table 3). Caregivers reported that 39.5% (*n* = 34) of children showed signs of anemia, 46.5% (*n* = 40) had feeding difficulties, and 55.8% (*n* = 48) showed signs of respiratory disease. Over three-quarters of the children were stunted (78.3%, *n* = 65/83), 8.8% (*n* = 7/80) were wasted, and 38.1% (*n* = 32/84) were underweight. Health and nutritional status did not vary significantly by age. Fifty-eight children (67.4%) scored below cut-off on the ASQ-3, and 29.1% (*n* = 25) of children were in the monitoring zone in at least one domain on the ASQ-3. Only 3.5% (*n* = 3) of children were considered to be developmentally on-track in all five developmental domains. There was no significant difference in developmental outcomes for children between 12 and 23 months (median ASQ-3 of 105, IQR: 70–165) and children over 24 months of age (median ASQ-3 of 142.5, IQR: 92.5–187.5, *p* = 0.11).

**Table 3:** Health, nutrition, and developmental status stratified by age

	Overall	12–23 Months	24+ Months	
	*n* = 86	*n* = 46	*n* = 40	*p*-value
Health Status				
Head Circumference (*n* = 84), n (%)				0.863
Normal	76 (90.5)	41 (91.1)	35 (89.7)	
Microcephaly	5 (6.0)	2 (4.4)	3 (7.7)	
Macrocephaly	3 (3.6)	2 (4.4)	1 (2.6)	
Caregiver-reported signs of anemia, n (%)				0.381
No	52 (60.5)	30 (65.2)	22 (55.0)	
Yes	34 (39.5)	16 (34.8)	18 (45.0)	
Caregiver-reported feeding difficulties, n (%)				1.000
No	46 (53.5)	25 (54.4)	21 (52.5)	
Yes	40 (46.5)	21 (45.7)	19 (47.5)	
Caregiver-reported signs of respiratory disease, n (%)				1.000
No	38 (44.2)	20 (43.5)	18 (45.0)	
Yes	48 (55.8)	26 (56.5)	22 (55.0)	
Nutritional Status^a^				
Stunting (*n* = 83), n (%)				1.000
Normal	18 (21.7)	10 (22.2)	8 (21.1)	
Moderate Stunting	37 (44.6)	20 (44.4)	17 (44.7)	
Severely stunting	28 (33.7)	15 (33.3)	13 (34.2)	
Wasting (*n* = 80), n (%)				0.407
Normal	73 (91.3)	40 (93.0)	33 (89.2)	
Moderate Wasting	6 (7.5)	2 (4.7)	4 (10.8)	
Severely wasting	1 (1.3)	1 (2.3)	0 (0.0)	
Underweight (*n* = 84), n (%)				0.323
Normal	52 (61.9)	29 (64.4)	23 (59.0)	
Moderately underweight	22 (26.2)	13 (28.9)	9 (23.1)	
Severely underweight	10 (11.9)	3 (6.7)	7 (18.0)	
Developmental Status on ASQ-3				
ASQ-3 Overall Sum Score, median (IQR)	120 (85–170)	105 (70–165)	142.5 (92.5–187.5)	0.113
ASQ-3 Developmental Status				0.604
On-track, n (%)	3 (3.5)	1 (2.2)	2 (5.0)	
Monitoring Zone, n (%)	25 (29.1)	15 (32.6)	10 (25.0)	
Delayed, n (%)	58 (67.4)	30 (65.2)	28 (70.0)	
Number of ASQ-3 Domains Delayed, median (IQR)	1 (0–2)	1 (0–2)	1 (0–2)	0.827

^a^Z-scores were not calculated for some children due to biologically infeasible values based on standard WHO Growth Standards scoring procedures

There was no association between ASQ-3 scores and the child’s sex (*p* = 0.161) or household socioeconomic status (*p* = 0.261; Table 4). Higher ASQ-3 scores were significantly associated with higher caregiver education with median ASQ-3 scores of 101 (IQR: 53–121), 136 (IQR: 93–188), and 193 (IQR: 143–225.5) among caregivers with no formal education, primary education, and secondary education, respectively (*p* = 0.001). Higher ASQ-3 scores were also significantly associated with having fewer other children in the household with median scores of 168 (IQR:121–176) among households with no other children, 131 (IQR:87.5–187) with one to three other children, and 104 (IQR:62.5–150) with four or more other children (*p* = 0.016).

**Table 4:** Bivariate Associations with Total Scores on the ASQ-3

	ASQ-3 Total Score	*p*-value
	Median (IQR)	
Demographic Characteristics		
Child’s sex		0.161
Male	130 (95–190)	
Female	105 (67.5–165)	
Education level		0.001
None	101 (53–121)	
Primary	136 (93–188)	
Secondary	193 (143–225.5)	
Socioeconomic Status		0.261
Category 2	119.5 (82.5–140.5)	
Category 3	133.5 (93–179)	
Unknown	132 (94–202)	
Other children in the household		0.016
No other children in the household	168 (121–176)	
1–3 other children in the household	131 (87.5–187)	
4 or more other children in the household	104 (62.5–150)	
Birth Characteristics		
Very low birth weight (*n* = 83)		0.043
Yes, < 1500 g	101 (77–121)	
No, > = 1500 g	135 (93–176)	
Size at Birth (*n* = 54)		
Small for gestational age	105 (60–137.5)	0.023
Not small for gestational age	151.5 (95–200)	
Prematurity Category (*n* = 55)		0.045
Very preterm (<32 weeks)	95 (85–160)	
Moderate/late preterm (32 to <37 weeks)	130 (95–170)	
Term (> = 37 weeks)	65 (15–90)	
Overall risk category (*n* = 54)		0.015
Term and small for gestational age	65 (15–90)	
Preterm and small for gestational age	115 (70–145)	
Preterm and not small for gestational age	151.5 (95–200)	
Health Status		
Head Circumference (*n* = 84)		0.004
Normal	120 (85–170)	
Microcephaly	0 (0–55)	
Macrocephaly	200 (165–235)	
Caregiver-reported signs of anemia		0.040
No	130 (87.5–200)	
Yes	107.5 (65–155)	
Caregiver-reported feeding difficulties		0.008
No	146.5 (90–200)	
Yes	100 (65–150)	
Caregiver-reported signs of respiratory disease		0.164
No	142.5 (70–205)	
Yes	112.5 (85–161.5)	
Nutritional Status		
Stunting (height/age, *n* = 83)		0.034
Normal	159 (102–176)	
Moderate	136 (103–203)	
Severe	104 (70.5–156.5)	
Wasting (weight/height, *n* = 80)		0.586
Normal	120 (85–175)	
Moderate/Severe^a^	130 (70–150)	
Underweight (weight/age, *n* = 84)		0.084
Normal	142.5 (99.5–183.5)	
Moderate	110 (87–169)	
Severe	99 (15.5–155)	

^a^Wasting is reported as normal or combined moderate and severe due to the small number of wasted children

ASQ-3 scores were also associated with children’s characteristics at birth; VLBW and SGA were significantly associated with lower ASQ-3 scores. VLBW children had median ASQ-3 scores of 101 (IQR: 77–121) compared to children born over 1500 g with median scores of 135 (IQR: 93–176, *p* = 0.043). Children born SGA had lower ASQ-3 scores (median = 105, IQR: 60–137.5) compared to those who were not SGA (median = 151.5, IQR:95–200, *p* = 0.023). Children born at term, with medical complications requiring admission to the neonatal unit, had significantly lower median ASQ-3 scores (median = 65, IQR: 15–90) than moderate/late preterm infants (median = 130, IQR: 95–170) and very preterm infants (median = 95, IQR: 85–160, *p* = 0.045). Term children who were SGA had the lowest ASQ-3 scores (median = 65, IQR: 15–90), with significant differences in scores from children who were preterm and SGA (median = 115, IQR: 70–145), and preterm but not SGA (median = 151.5, IQR: 95–200, *p* = 0.015).

Children’s head circumference was significantly associated with ASQ-3 scores; children with microcephaly had significantly lower ASQ-3 scores (median = 0, IQR: 0–55) than children with normal head circumference (median = 120, IQR: 85–170, *p* = 0.004). Children with reported anemia symptoms and children with feeding difficulties had lower ASQ-3 scores (median = 107.5, IQR: 65–155, and median = 100, IQR: 65–150, respectively) than children who did not (median = 130, IQR: 87.5–200, *p* = 0.040, and median = 146.5, IQR: 90–200, *p* = 0.008, respectively). There was no association between respiratory symptoms and ASQ-3 scores (*p* = 0.164). A significant association was found between stunting and lower ASQ-3 scores, with median ASQ-3 scores of 159 (IQR: 102–176), 136 (IQR: 103–203), and 104 (IQR: 70.5–156.5) for normal, moderately, and severely stunted children, respectively (*p* = 0.034). There was no association with wasting (*p* = 0.586) or underweight (*p* = 0.084) and ASQ-3 scores.

## Discussion

Infants born PT/LBW in rural Rwanda had high rates of abnormal reported health status, undernutrition, and potential abnormal development at one to three years of life, indicating a key service delivery gap for this vulnerable population. This study has a number of key findings.

First, caregivers reported high rates of abnormal symptomatic health status. Approximately one-half of caregivers reported signs of anemia, feeding difficulties and respiratory disease in their children. While caregiver-reported child health status results must be interpreted with caution, as they are not verified by clinician diagnosis, these symptoms are all known potential sequelae of prematurity [7, 8]. However, it was reassuring to find low rates of abnormal head size among those alive and assessed.

Second, children born PT/LBW also had high rates of undernutrition. Stunting was seen in these children at rates nearly double the national stunting prevalence of 41% among rural Rwandan children [17]. Levels of wasting and underweight were more than triple the Rwandan rural prevalence (2% wasted and 10% underweight nationally) among children under-five [17]. These results could have been compounded by the high rates of reported health problems [33]. Furthermore, poor maternal nutrition, a main contributor to IUGR, is also important to note as nearly half of the children were SGA at birth.

Third, and most importantly, the number of children falling below the cut-off on the ASQ-3 or in the monitoring zone was high. Rates of poor ASQ-3 screening were higher in this sample than among other children of similar ages in rural Rwanda who were not necessarily preterm or born at low birth weight, which showed between 10.3 and 35.1% of children falling below domains on the ASQ-3 [31]. This indicates a clear need for early intervention services to ensure delays are addressed promptly and prevent potential long-term challenges. Our findings support other studies that used the ASQ to assess development in diverse settings and found that preterm infants are at high-risk of delay [15, 34]. While the first 2 years of life are a period with the potential for substantial catch-up, this study found similarly abnormal ASQ-3 results in children aged 24 months or older as those 12–23 months, highlighting a clear need for services to help children born preterm, LBW, and growth restricted to reach their developmental potential.

A number of factors were associated with slower development. Stunting was significantly linked with lower ASQ-3 scores; as the child’s severity of stunting increased, scores on the ASQ-3 decreased. This is consistent with other studies demonstrating the link between chronic malnutrition and impaired early childhood development [5, 35]. Fortunately, stimulation-focused interventions have proven effective in helping stunted children achieve improvements in cognitive development [36]. Concurrent interventions in the immediate postnatal period to support catch-up growth and stimulation are essential to help PT/LBW infants reach normal growth to prevent developmental impairments associated with chronic undernutrition [37].

Children who were SGA or VLBW had worse developmental scores than children who were born at a normal size for gestational age or at higher weight. These findings support research from high-income countries showing that IUGR and VLBW are associated with worse developmental outcomes [4]. Developmental delay estimates for ELBW infants from high-income countries ranges from 20 to 65% [38]. Our study found similarly high rates of abnormal development among VLBW and LBW infants. Contrary to other studies [15, 34], term infants in this sample had significantly lower ASQ-3 scores than preterm infants. However, these were not healthy term infants as they were SGA and likely had other medical comorbidities not documented for this study that required admission to the neonatal care unit.

While no association was found with socioeconomic status using Rwanda’s *ubudehe* classification and developmental outcomes, the sample in this study was very poor overall which may have contributed to the null findings. However, the strong associations of lower caregiver education and a greater number of children in the household with lower developmental scores are consistent with other studies demonstrating that poverty is associated with worse developmental outcomes [39]. Healthy child development requires an environment with access to adequate resources such as food, parental interaction, and stimulation [39]. Households with high poverty and low education often have more limited opportunities for stimulation [40] and higher rates of malnutrition [41]. These compounded adversities hinder children’s ability to reach their developmental potential and require interventions to address all the drivers of poor developmental outcomes [42].

This study has some limitations. As a cross-sectional descriptive study of an at-risk population, there is no comparison of developmental data for the general population so associations can be described but causality could not be assessed. Another potential limitation is that no tool for measuring development has been validated with local cut-points in Rwanda; however the ASQ-3 has been adapted and used in recent studies in Rwanda [31, 43]. Because there is no Rwandan norm, we used cut-points and continuous scores, similar to other studies using the ASQ-3 in sub-Saharan Africa [28, 29]. The use of routinely collected data from the hospital also posed some challenges with data quality, particularly for missing gestational age data which prohibited using adjusted age for developmental and nutritional assessment. Adjusted age is typically used for children under 24 months who are more than 3 weeks premature [32]. However, we found no significant difference in ASQ-3 scores based on children’s age demonstrating continued concern even after preterm children would have been expected to catch up.

The large number of children who could not be traced may lead to underestimation of mortality, malnutrition, and abnormal development in this study. These children were different from the children included in the household survey portion of this study. Children unable to be located for household data collection were significantly more premature than the located children and therefore at a higher mortality risk [2, 3, 12]. Overall, neonatal mortality in the district of Rwanda where this study took place is estimated to be 35 per 1000 live births and post-neonatal infant mortality is 26 per 1000 live births, both of which are high and infants born preterm are at greater risk of mortality during this period [17]. Further, a study from three rural district hospitals in Rwanda, including Rwinkwavu District Hospital, found a that 29.5% of infants who were VLBW or very preterm died prior to discharge from the neonatal care unit [44]. Lastly, multivariate analysis for predictors of developmental outcomes was not feasible due to the small sample and non-normal distribution of ASQ-3 scores. Despite limitations, this study contributes important findings to the limited literature on the outcomes among children born PT/LBW in rural sub-Saharan Africa.

## Conclusions

This study found high rates of abnormal health status, undernutrition, and impaired development among PT/LBW children discharged from a hospital neonatal unit in rural Rwanda. Early intervention, the standard of practice in developed countries [10]. is essential for these at-risk children. Services that support caregivers to promote positive parenting, cognitive stimulation, and improved nutrition could help at-risk children achieve developmental milestones and are lacking across sub-Saharan Africa [45]. As efforts intensify to improve survival of PT/LBW infants globally, our findings have significant implications for policy and service delivery to support these children to thrive. There is an urgent need to invest in programs at scale to support the growing number of PT/LBW infants surviving across sub-Saharan Africa and unlock their developmental potential.

## Additional file

Additional file 1: The data collection tool, excluding the copyrighted Ages and Stages Questionnaires (ASQ-3), which are only available from Paul H. Brookes Publishing Co., is available in the supplementary materials as follows: This is the study questionnaire that was used for data collection. (DOCX 22 kb)

## Abbreviations

ASQ-3: Ages and Stages Questionnaires, version 3
ELBW: Extremely low birth weight
IUGR: Intrauterine Growth Restriction
LBW: Low birth weight
PIH/IMB: Partners In Health/Inshuti Mu Buzima
PT: Preterm
SGA: Small for gestational age
VLBW: Very low birth weight
