# Supplementary material for: Health, nutrition, and development of children born preterm and low birth weight in rural Rwanda: a cross-sectional study
Source: BMC Pediatr. 2017 Nov 15;17:191. doi: 10.1186/s12887-017-0946-1 (PMC5688768; doi:10.1186/s12887-017-0946-1)
Supplement: Supplementary file 1 — The data collection tool, excluding the copyrighted Ages and Stages Questionnaires (ASQ-3), which are only available from Paul H. Brookes Publishing Co., is available in the supplementary materials as follows: This is the study questionnaire that was used for data collection. (DOCX 22 kb) [file 12887_2017_946_MOESM1_ESM.docx]

**Data Collection Tool for “Health, Nutrition, and Development of Children Born Preterm and Low Birth Weight in Rural Rwanda: a Cross-sectional Study” by Kirk et al.**

**Neonatal Medical File Data:**

Infant Name:

Mother/Caregiver’s name:

Infant Sex: ☐ Male ☐ Female

District:

Sector:

Cell:

Village:

Health Center: ____________

Birth Date: _______________

Gestational Age: __________

Birth Weight: _____________

**For household data collection:**

**Demographics:**

1. Who is the primary caregiver for the child?

☐ Mother ☐ Father ☐ grandmother ☐ sister

☐ Other (specify):_______________________

1. What is your current marital status?

☐ Married ☐ Cohabiting ☐ Single ☐ Divorced ☐ Widowed

1. What is the highest level of education you have completed?

☐ None ☐ Primary ☐ Secondary ☐ University

1. How many years of school have you completed? ______________
2. What is your ubudehe category (measure of economic status)?

☐ Group 1 ☐ Group 2 ☐ Group 3 ☐ Group 4

☐ Group 5 ☐ Group 6 ☐ Unknown/ not applicable

1. How many living children in the home? _______________

**Medical Complications:**

1. At any point in your child’s life, were you ever told by a doctor or nurse that your child’s head was abnormally large, which may have required medical care?

☐ Yes ☐ No

1. Does your child appear pale in the eyes, or weak, or past history of blood transfusions?

☐ Yes ☐ No

1. Does your child have trouble eating, such as choking, coughing or gagging while eating?

☐ Yes ☐ No

1. Does your child have difficulty breathing on a daily basis, such as breathing fast, or chronic cough, or out of breath when walking?

☐ Yes ☐ No

**Growth and Nutrition**:

Current Weight (kg): ___________ Z-score (Height/Age): _________

Current Height (cm): __________ Z-score (Weight/Age):________

Current Head Circumference (cm):_______ Z-score (Weight/Height): ________

MUAC (cm): ___________

**Developmental Milestones**:

Developmental Screening Tool: Age-appropriate Ages and Stages Questionnaires^[[1]](#footnote-1)^ version 3 (ASQ-3), which are copyrighted and available from Paul H. Brookes Publishing Co.

#

# Urupapuro rw ’ikusanyamakuru: Gukurikirana ubuzima bw’umwana kuva avutse

Izina ry’umwana:____________________

Izina rya nyina/umurezi:_____________________

Igitsina cy’umwana

Akarere:

Umurenge:

Akagari:

Umudugudu:

Ikigo nderabuzima:______________

Itariki y’amavuko:

Ibyumweru yavukiyeho:_______________

Ibiro yavukanye:_____________________

Uyu mwana: ☐ Ariho ☐ yitabye imana ☐ yaburiwe irengero

**Amakuru y’irangamimerere n’amakuru y’ingenzi areba urera umwana**

1. Ni rihe sano ufitanye n’umwana:

☐ Nyina ☐ umurezi ☐Nyirakuru ☐ mushiki we/ mukuru we

☐ undi ­­­­­­­­­­­­­­(sobanura)_______________________

1. Irangamimirere:

☐yashatse byemewe n’amategeko ☐ Babana bitemewe n’amategeko ☐ingaragu ☐baratandukanye ☐yarapfakaye

1. Ni uruhe rwego rwa nyuma rw’amashuri wagezeho:

☐Ntabwo yize ☐amashuri abanza ☐amashuri yisumbuye ☐kaminuza

1. Warangije imyaka ingahe y’amashuri:_________
2. Ubarizwa mu cyihe cy’ubudehe: ☐ icyiciro cya1 ☐ icyiciro cya 2 ☐ icyiciro cya 3 ☐ icyiciro cya 4 ☐ icyiciro cya 5 ☐ icyiciro cya 6 ☐ ntikizwi
3. Ese ubana n’ abana bangahe mu rugo?___________

**Ingaruka ku buzima bw’umwana**

1. Hari igihe waba warigeze ubwirwa n’umuganga cg umoforomo ko umutwe w’umwana wawe ari munini bidasanzwe ku buryo bikeneye kuvurwa? ☐ Yego ☐ Oya
2. Ese ubona Umwana wawe afite amaso yeruruka, acika intege, nke cyangwa yaba yarigeze kongererwa amaraso? ☐ Yego ☐ Oya
3. Ese umwana wawe yaba agira ibibazo arimo kurya nko: kunigwa n’ibyo kurya,gukorora cyangwa kwihaga ? ☐ Yego ☐ Oya
4. Ese umwana wawe ajya agira ibibazo byo guhumeka buri gihe nko guhumeka vuba na vuba, inkorora ihoraho, cyangwa kubura umwuka arimo kugenda? ☐ Yego ☐ Oya

**Imikurire n’imirire**

Ibiro afite (kg):__________ Igipimo cya Z (W/A):_____________

Uburebure afite (cm):_________ Igipimo cya Z(H/A):___________

Umuzenguruko w’umutwe we (cm):_____________ Igipimo cya Z (W/H):__________

Umuzenguruko w’akaboko ke (cm):______________

**Ibijyanye n’imikurire**
Igipimo cy’imikurire y’umwana (reba ku mugereka D).

1. Squires, J., Twombly, E., Bricker, D., & Potter, L. (2009). *ASQ-3 User's Guide*. Baltimore: Paul H. Brookes Publishing Company. [↑](#footnote-ref-1)
